# Editorial: Considering plant metabolites and their synthetic derivatives as candidates for the development of drugs against multidrug resistant (MDR) tumors

**DOI:** 10.3389/fphar.2022.1108252

**Published:** 2022-12-07

**Authors:** Patricia Rijo, Constantinos M. Athanassopoulos, María Cecilia Carpinella

**Affiliations:** ^1^ CBIOS—Universidade Lusófona Research Center for Biosciences & Health Technologies, Lisbon, Portugal; ^2^ Faculty of Pharmacy, Instituto de Investigação do Medicamento (iMed.ULisboa), University of Lisbon, Lisbon, Portugal; ^3^ Synthetic Organic Chemistry Laboratory, Department of Chemistry, University of Patras, Patras, Greece; ^4^ Fine Chemical and Natural Products Laboratory, IRNASUS CONICET-UCC, School of Chemistry, Universidad Católica de Córdoba, Córdoba, Argentina

**Keywords:** plant-derived compounds, synthetic derivatives, multidrug resistant (MDR) tumors, cancer therapy, phytochemicals

Despite the great benefits of chemotherapy, the ability of tumor cells to acquire cross-resistance to multiple antineoplastic drugs (MDR) results in a drastic reduction in the efficacy of these agents. Of greatest concern is the consequent failure in clinical practice, which remains one of the major barriers to successful cancer treatment. To improve patient outcome, novel efficacious anti-cancer agents or approaches to reverse MDR are necessary.

Plants have been an endless and vital source of pharmacologically active compounds, many of which are widely used in cancer therapies, not only to control tumor cells, but also to increase their sensitivity to chemotherapeutic agents. The structural diversity and array of biological properties of plant-derived metabolites, in particular against tumors, make these strong candidates to combat MDR cells or establish their scaffolds as a valuable base for the synthesis of derivatives.

This enormous potential of plants to tackle MDR tumors encouraged us to publish this Research Topic to display and discuss the cutting-edge research in the field. As editors, it was a pleasure to review a wide range of fascinating articles with different perspectives, and we summarize here the main findings of accepted articles.

In search of compounds with anti-cancer properties, Zhang et al. investigated the mechanism by which the anthraquinone emodin, isolated from *Rheum palmatum*, *Polygonum cuspidatum* and *Aloe vera*, affects the breast cancer cells, MCF-7. The compound was able to activate the aryl hydrocarbon receptor (AhR), with a subsequent increase in expression of the cytochrome P450 1A1 (CYP1A1), which led to inhibition of proliferation of the MCF-7 cell line and induced apoptosis. These results show the potential of emodin as an alternative agent to treat resistant breast cancer.

The role of the epidermal growth factor receptor (EGFR) vIII in glioblastoma multiforme (grade IV) erlotinib-associated resistance led Powe et al. to evaluate the effect of combining this drug with the flavonoid luteolin on this target. They determined that co-administration of both agents to the glioblastoma cells diminished cell proliferation, induced cytotoxicity, and altered the cell morphology. The combined compounds increased the expression of cleaved PARP and cleaved caspase, downregulated Bcl-xL and upregulated BAD, resulting in augmented apoptosis compared to separate treatments with each agent. In addition, the combination of erlotinib and luteolin significantly downregulated the phosphorylated EGFR cell signaling proteins Akt, mTOR, NF kappa B and STAT3. These findings suggest that the combination of the EGFR inhibitor erlotinib with luteolin may offer a promising strategy to overcome glioblastoma resistance.

Llorens de los Ros et al. screened a panel of extracts from 40 Argentinian plants for the inhibition of angiogenesis and characterized α-terthienylmethanol as the active principle causing the potent anti-angiogenic activity observed with *Tagetes minuta*. The underlying mechanism of action was shown to be the terthiophene inhibiting the protein kinase C (PKC) isozymes α and β2. A detailed molecular dynamic simulation showed that the subject compound mimics both the binding site and the dynamic behavior of the crystallographic inhibitors NVP-AEB071 for PKC-α, and sotrastaurin for PKC-β. This research is a valuable contribution for the rational design of novel inhibitors of angiogenesis inspired in the terthiophene scaffold, which could provide innovative tools against MDR tumors.

Magalhes et al. describe the potential therapeutic use of the compound, parvifloron D (ParvD), isolated from *Plectranthus ecklonii* to treat glioblastoma. They focused on a natural lead drug that induced G2/M cell cycle arrest and apoptosis *via* activation of the intrinsic mitochondria-dependent pathway. The doses of ParvD necessary to induce pronounced inhibitory effects were substantially lower than those needed for temozolomide (TMZ, first-line treatment) to give comparable effects. ParvD may have the potential to overcome the resistance related to TMZ and position itself as a lead drug for future chemotherapeutics.

All these findings may pave the way to improve outcomes and benefit patients with drug-resistant cancers.

